# Child and adolescent bicycling injuries involving motor vehicle collisions

**DOI:** 10.1186/s40621-019-0185-z

**Published:** 2019-03-04

**Authors:** Tona M. Pitt, Alberto Nettel-Aguirre, Gavin R. McCormack, Andrew W. Howard, Camilla Piatkowski, Brian H. Rowe, Brent E. Hagel

**Affiliations:** 1 Department of Community Health Sciences, Cumming School of Medicine, University of Calgary, 3330 Hospital Dr NW, Calgary, T2N 4N1 Canada; 2 Department of Paediatrics, Cumming School of Medicine, University of Calgary, 28 Oki Drive NW, Calgary, T3B 6A8 Canada; 3 Alberta Children’s Hospital Research Institute, Cumming School of Medicine, University of Calgary, 28 Oki Drive NW, Calgary, T3B 6A8 Canada; 4 Faculty of Environmental Design, University of Calgary, PF 2182, 2500 University Dr NW, Calgary, AB T2N 1N4 Canada; 5 Departments of Surgery and Health Policy, Management & Evaluation, University of Toronto, 555 University Ave, Toronto, M5G 1X8 Canada; 6 O’Brien Centre for the Bachelor of Health Sciences, Cumming School of Medicine, University of Calgary, 3330 Hospital Dr NW, Calgary, T2N 4N1 Canada; 7 Department of Emergency Medicine and School of Public Health, University of Alberta, 8303 - 112 Street, Edmonton, T6G 2T4 Canada

**Keywords:** Youth injury, Bicycle collisions, Severe bicycle injuries, Police reports, Classification tree analysis

## Abstract

**Background:**

Bicycle-related injuries are among the most common recreational injuries for children in Canada; moreover, bicycle-motor vehicle collisions often result in serious injuries. This study seeks to examine environmental, motorist, and bicyclist characteristics of bicycle-motor vehicle collisions that resulted in police reported severe injuries in youth (< 18 years old) bicyclists, in Alberta, Canada.

**Findings:**

Using Calgary and Edmonton police collision reports, 423 youth bicycle-motor vehicle collisions were identified from 2010 to 2014. Forty-three (10.2%) of these collisions resulted in major/fatal (severe) injuries. These severe injury cases were compared with the 380 youth bicycle-motor vehicle collisions resulting in minor or no injury (controls) using classification tree and logistic regression analyses. There were no driver or bicyclist characteristics with a significant effect on the odds of severe injury to youth bicyclists; however, lower odds were found on each of: divided roads with no barrier (aOR = 0.36; 95% CI: 0.13–0.97) or during peak traffic time (aOR = 0.44; 95% CI: 0.16–0.99).

**Conclusion:**

Personal and environment characteristics should be considered in future research and interventions focused on reducing severe youth bicycle-motor vehicle collision injuries.

**Electronic supplementary material:**

The online version of this article (10.1186/s40621-019-0185-z) contains supplementary material, which is available to authorized users.

## Introduction

Bicycling has physiological and environmental benefits (de Hartog et al. 2010). However, bicycling-related injuries are one of the most common causes of hospitalization for youth (< 18 years old) in Canada (Canadian Institute for Health Information 2017). The risk of injury, particularly due to motor-vehicle collision, is a major deterrent to bicycling for both parents and youth (Jacobsen et al. 2009). Children and adolescents have less developed cognitive ability, poorer situational awareness and worse hazard perception than adults, potentially increasing risk for bicycling injury (Lehtonen et al. 2017; Barton and Morrongiello 2011).

A recent systematic review of severe bicycling injury literature concluded that studies to date tend to focus on cyclists admitted to hospital or presenting in emergency departments (ED) (Embree et al. 2016). Data collection in these institutional settings focused on the youth cyclist and their behaviour, and that while previous work indicates there is a higher risk of severe injury in youth cyclists when a motor-vehicle is involved (Hagel et al. 2015; Mitchell et al. 2015), there is no information regarding the characteristics of the motorists involved (e.g., age, sex, vehicle). Moreover, this review highlighted the need for research comparing those bicyclists who are severely injured with those who are not severely injured (Embree et al. 2016).

One approach to comparing severely injured cyclists with non-severely injured is to employ police collision reports. Police reports contain a spectrum of injury severities as those involved may not necessarily require medical attention to be included. It is important to keep in mind that although these data have the potential to include such non-injurious events, there is a possibility of misclassification where those who do not have injury initially may have injury progress in the hours or days following the collision. This dataset also captures motorists and environmental characteristics in addition to youth characteristics for bicycle-motor vehicle collisions (BMVCs). The aim of this study was to examine the environmental, motorist and youth characteristics of youth BMVCs by comparing collisions where the cyclist was reported by police to have severe injury with those who have non-severe injury, in urban environments located in Alberta, Canada. It is important to understand how these characteristics are contributing to the most severe injuries to improve current and future primary prevention strategies. Specifically, we chose to examine the cities of Calgary and Edmonton. These two cities are the largest in Alberta and during the time of study had populations of approximately 1,265,531 and 928,182, respectively (Statistics Canada 2012). The City of Calgary, in 2010, had approximately 712 km of multi-use pathways and 355 km of on-street bikeways (City of Calgary 2011), while Edmonton had “160 km of multi-use pathways, 100 km of shared pathways and 105 km of street routes” (Government of Alberta 2012). To improve winter ridership, policies have been implemented to improve the maintenance of existing cycling structure (City of Calgary 2011; City of Edmonton 2009). Both cities primarily use private vehicles for transportation; approximately 79 and 77.6% of all trips use private vehicles in Calgary and Edmonton, respectively (City of Calgary 2013; City of Edmonton 2015).

## Methods

This study uses detailed police traffic collision reports from 2010 to 2014 in Edmonton and Calgary, Alberta that have been digitized. The Alberta police collision reports are consistent across the two municipalities. Youths riding a bicycle at the time of a collision with a motor vehicle that was not parked were included as BMVCs. Severe injury cases were defined as collisions resulting in police reported ‘Major Injury’ (admitted to hospital) or ‘Fatal Injury’ to the cyclist. Non-severe injury controls were the remaining collisions where cyclist sustained ‘Minor Injury’ (treated and/or released from an ED) or ‘No Injury.’ As mentioned, there is a possibility that those with ‘No Injury’ may progress to having injury following the collision, and this may be further complicated if the officer does not follow up with the bicyclist to confirm injury severity. To this end, we believe that the combination of ‘Minor Injury’ and ‘No Injury’ may mitigate this potential bias to some degree.

Logistic regression was used to estimate crude and adjusted odds ratios (ORs, aORs) for severe injury of the youth bicyclist compared with non-severe injury using STATA v.12.1 (STATA Corp 2011). As sample size was small with 43 cases, we chose to perform an exploratory analysis using Classification and Regression Trees (CART). This is because traditional methods of statistically driven variable selection (for example purposeful selection) may be underpowered to detect statistical inclusion of variables and the use of backward selection, for example, may overfit the model given the smaller sample of cases. To this end, we chose to explore what variables were potentially important using CART (CART implemented using the recursive partitioning rpart v.4.1.2 package in R software (R Core Team 2018)). We then used these variables to inform multivariable logistic regression models, which provided us with estimates of odds ratios and adjusted odds ratios. CART analysis is described in detail elsewhere (Breiman et al. 1984); however, briefly, it uses an algorithm to split the original dataset into subgroups to generate less heterogeneous groups at the node resulting from each split (i.e., proportion of outcome) within a variable. This process is carried out within subgroups until the model fails to improve according to a given complexity parameter or the final nodes have reached the minimum amount of observations allowed for a further split, hence becoming a terminal node (leaves). A similar analysis using decision tree analysis has been used previously in all age bicycle crashes (Prati et al. 2017).

For this analysis, the complexity parameter and minimum node size were adjusted to ensure that the tree was large enough to understand what exposure variables were influential to the outcome, without the tree becoming so large that the model was overfitting. After growing the tree, the variables that contributed to separating subgroups regarding the outcome (case or control) were identified. Moreover, the recursive portioning package used in this analysis has in itself a 10-fold cross validation for the growth/size of the tree. It is important to note that given sample size limitations, we were unable to use CART as a predictive model and therefore do not include a training set. Rather, we chose to use CART as an exploratory analysis of the data that may help inform logistic regression models. Three logistic regression models comparing cyclist, motorist, and environmental characteristics were built based on the CART results (included as Additional file 1: Figure S1). As well, the model examining the environmental characteristics was further adjusted by cyclist age and sex, as these were previously observed risk factors for severe bicyclist injury (Martinez-Ruiz et al. 2013; Tin Tin et al. 2010) and may confound results as they relate to the built environment (Romanow et al. 2012).

The variables considered for each model are presented with crude odds; those without an adjusted estimate were not identified in by the exploratory CART analysis and were therefore not included in the logistic regression modelling. Briefly, the variables included came from the police collision reports and are therefore, reliant on the officer’s report of the circumstances surrounding the collision. While many of the police collision report variables and the way by which they are split are self-explanatory (i.e., sex), some may be less commonly used. For example, the unsafe speed variable in this situation does not represent a raw measure of speed (as this is generally unavailable) but indicates if, based on the judgement of the officers, the speed of the vehicle was too great for the given conditions. Peak time represents a proxy for times where traffic volume is expected to be highest, defined as 6:00 am-8:59 am or 4 pm-5:59 pm on weekdays (Romanow et al. 2012). The environmental factors such as driving condition and light condition are separated into normal and poor, where poor is a combination of less desirable conditions (sun glare or darkness for light condition or any combination of wet, snow/slush, construction or loose surface materials for driving condition). The combinations of such variables were made as each one may only contribute one or two instances but represent the overall notion that the driving situation was not in clear conditions.

## Results

Overall, 423 youth BMVCs were identified; 43 (10.2%) were severe injury cases. The CART exploration identified age (categorized into thirds), sex, driver action, and impact location of the motorist and age, sex and cyclist action in the cyclist as important to severe injury. CART analysis identified peak traffic times, traffic control device, road type, driving conditions and intersection status as important in the environmental characteristics model. For logistic regression models, there were no significant driver or cyclist variables associated with severe injury after BMVCs (Table 1 and Table 2). Environmental characteristics occurring during peak traffic time (aOR = 0.40; 95% CI: 0.16–0.99) or on divided roads with no physical barrier (aOR = 0.36; 95% CI: 0.13–0.97) were associated with lower odds of severe injury (Table 3).

**Table 1:** Comparison of characteristics between youth sustaining severe and non-severe injuries after BMVC

Bicyclist Factors	Collision resulting in severe injuries (%)	Collision resulting in non-severe injuries (%)	Unadjusted odds ratio (95% CI)	Adjusted odds ratio ^a^(95% CI)
	*n* = 43	*n* = 380		
Bicyclist Action				
Driving Properly	9 (20.9)	100 (28.2)	1.00	1.00
Failure to Yield at Uncontrolled Intersection	4 (9.3)	28 (7.9)	1.59 (0.45–5.54)	1.35 (0.38–4.77)
Traffic Control Device Violation	3 (7.0)	50 (15.2)	0.67 (0.17–2.57)	0.69 (0.18–2.67)
Other (Improper turn/lane change etc.)	7 (16.3)	49 (13.8)	1.59 (0.56–4.51)	1.53 (0.53–4.42)
Unknown	20 (46.5)	153 (36.1)	1.45 (0.64–3.32)	1.39 (0.60–3.21)
Age (Years)				
< 7	5 (11.6)	27 (7.1)	1.00	1.00
7 to 12	19 (44.2)	139 (36.3)	0.74 (0.25–2.14)	0.83 (0.28–2.48)
13–17	19 (44.2)	214 (56.3)	0.48 (0.17–1.39)	0.53 (0.18–1.55)
Sex				
Female	4 (9.3)	75 (19.7)	1.00	1.00
Male	39 (90.7)	305 (80.3)	2.39 (0.83–6.92)	2.35 (0.81–6.85)
Helmet				
Wearing Helmet	14 (32.6)	147 (38.7)	1.00	–
Not Wearing	25 (58.1)	183 (48.2)	1.43 (0.72–2.86)	–
Unknown	4 (9.3)	50 (13.2)	0.84 (0.26–2.67)	–
Unsafe Speed				
No	22 (51.2)	189 (49.7)	1.00	–
Yes	2 (4.7)	20 (5.3)	0.86 (0.19–3.92)	–
Unknown	19 (44.2)	171 (45.0)	0.95 (0.50–1.82)	–

*CI* confidence interval

^a^Adjusted for Bicyclist Action, Age and Sex

**Table 2:** Comparing driver characteristics in youth BMVCs resulting in severe vs. non-severe injuries to cyclist

Driver Factors	Collision resulting in severe injuries (%)	Collision resulting in non-severe injuries (%)	Unadjusted odds ratio (95% CI)	Adjusted odds ratio^a^ (95% CI)
	*n* = 43	*n* = 380		
Driver Action				
Driving Properly	19 (44.2)	179 (47.1)	1.00	1.00
Failure to Yield at Uncontrolled Intersection	7 (16.3)	67 (17.6)	0.98 (0.40–2.45)	0.87 (0.33–2.25)
Traffic Control Device Violation	2 (4.7)	18 (4.7)	1.05 (0.23–4.86)	1.06 (0.21–5.23)
Backed Vehicle Unsafely	4 (9.3)	11 (2.9)	3.42 (0.99–11.82)	6.61 (0.51–86.6)
Other (Improper turn/lane change etc.)	7 (16.3)	29 (7.6)	2.27 (0.88–5.88)	1.97 (0.72–5.38)
Unknown	4 (9.3)	76 (20.0)	0.50 (0.16–1.51)	0.51 (0.15–1.66)
Age				
16 to 24	7 (16.3)	39 (10.3)	1.71 (0.63–4.64)	1.81 (0.62–5.03)
25 to 39	11 (25.6)	105 (27.6)	0.99 (0.42–2.35)	1.06 (0.44–2.58)
40 to 54	12 (28.0)	114 (30.0)	1.00	1.00
55 to 91	10 (23.2)	73 (19.2)	1.30 (0.53–3.17)	1.17 (0.46–2.99)
Unknown	3 (7.0)	49 (12.9)	0.58 (0.16–2.15)	0.69 (0.08–6.03)
Impact Location				
Front Centre	30 (69.8)	206 (54.2)	1.00	1.00
Back	4 (9.3)	15 (3.9)	1.83 (0.57–5.89)	0.36 (0.03–4.71)
Left Side	2 (4.7)	48 (12.6)	0.29 (0.07–1.24)	0.22 (0.05–1.01)
Right Side	7 (16.3)	90 (23.7)	0.53 (0.23–1.26)	0.53 (0.22–1.29)
Unknown	0 (0.0)	21 (5.5)	–	–
Sex				
Female	13 (30.2)	160 (42.1)	1.00	1.00
Male	28 (65.1)	192 (50.5)	1.79 (0.90–3.58)	1.82 (0.90–3.76)
Unknown	2 (4.7)	28 (7.4)	0.88 (0.19–4.11)	2.36 (0.17–32.32)
Unsafe Speed				
No	25 (59.5)	253 (66.8)	1.00	–
Yes	2 (4.8)	8 (2.1)	2.53 (0.51–12.57)	–
Unknown	16 (35.7)	119 (31.1)	1.36 (0.70–2.64)	–
Vehicle Type				
Passenger Car	18 (41.9)	203 (53.4)	1.00	–
Truck/Van/SUV	20 (46.5)	156 (41.1)	1.45 (0.74–2.83)	–
Commercial Vehicle	3 (7.0)	16 (4.2)	2.11 (0.56–7.95)	–
Unknown	2 (4.7)	5 (1.3)	4.51 (0.82–24.92)	
Alcohol Use				
Impaired by Alcohol	0 (0.0)	1 (0.3)	–	–
Apparently Normal	38 (88.4)	313 (82.4)	–	–
Unknown	5 (11.63)	66 (17.4)	–	–

*CI* confidence interval, *SUV* sport utility vehicle

^a^Adjusted for Driver Action, Age, Impact Location and Sex

**Table 3:** Comparison of external factors in BMVCs resulting in severe or non-severe injuries to youth bicyclists

Environmental Factors	Collision resulting in severe injuries (%)	Collision resulting in non-severe injuries (%)	Unadjusted odds ratio (95% CI)	Adjusted odds ratio^b^ (95% CI)	Adjusted odds ratio^c^ (95% CI)
	*n* = 43	*n* = 380			
Driving Conditions					
Normal	42 (97.7)	357 (93.9)	1.00	1.00	1.00
Poor	1 (2.3)	23 (6.1)	0.37 (0.05–2.81)	0.38 (0.05–3.00)	0.40 (0.05–3.26)
Intersection					
Yes	26 (60.5)	252 (66.3)	0.77 (0.41–1.48)	1.27 (0.50–3.21)	1.38 (0.55–3.54)
No	17 (39.5)	128 (33.7)	1.00	1.00	1.00
Peak Traffic Time^a^					
Yes	6 (14.0)	114 (30.1)	**0.38 (0.15–0.91)**	**0.37 (0.15–0.91)**	**0.40 (0.16–0.99)**
No	37 (86.0)	264 (69.9)	1.00	1.00	1.00
Unknown	0 (0.0)	2 (4.7)	–	–	–
Road Type					
Divided w/ Barrier	19 (44.2)	138 (36.3)	1.00	1.00	1.00
Divided No Barrier	6 (14.0)	112 (29.5)	0.39 (0.15–1.01)	**0.37 (0.14–0.99)**	**0.36 (0.13–0.97)**
Undivided Two-Way	8 (18.6)	66 (17.4)	0.88 (0.70–2.12)	0.72 (0.25–2.15)	0.63 (0.20–1.95)
Undivided One-Way	2 (4.7)	8 (2.1)	1.82 (0.39–9.19)	1.73 (0.31–9.53)	1.89 (0.34–10.55)
Other	8 (18.6)	56 (14.7)	1.04 (0.43–2.51)	0.80 (0.23–2.74)	0.70 (0.20–2.48)
Traffic Control Device					
Nothing Present	21 (48.8)	156 (41.1)	1.00	1.00	1.00
Crosswalk	10 (23.2)	59 (15.5)	1.26 (0.56–2.83)	1.13 (0.41–3.18)	1.13 (0.39–3.25)
Traffic Lights	7 (16.3)	95 (25.0)	0.55 (0.22–1.34)	0.46 (0.14–1.46)	0.45 (0.14–1.49)
Sign Present	3 (7.0)	62 (16.3)	0.36 (0.10–1.25)	0.34 (0.08–1.39)	0.32 (0.08–1.34)
Unknown/Other	2 (4.7)	8 (2.1)	1.86 (0.37–9.34)	1.50 (0.27–8.34)	1.73 (0.29–10.38)
Hit and Run					
Yes	3 (7.0)	52 (13.7)	0.47 (0.14–1.59)	–	–
No	40 (93.0)	328 (86.3)	1.00	–	–
Light Condition					
Normal	39 (89.5)	340 (89.5)	1.00	–	–
Poor (Sunglare/Darkness)	4 (10.5)	34 (8.9)	1.02 (0.35–3.04)	–	–
Unknown	0 (0.0)	6 (1.6)	–	–	–
Road Curve					
Straight	21 (48.8)	155 (40.8)	1.00	–	–
Curved	5 (11.6)	17 (4.5)	2.17 (0.73–6.50)	–	–
Unknown	17 (39.5)	208 (54.7)			
Road Grade					
Flat	25 (58.1)	157 (41.3)	1.00	–	–
Graded	1 (2.3)	16 (4.2)	0.39 (0.05–3.09)	–	–
Unknown	17 (39.5)	207 (54.5)	**0.52 (0.27–0.98**)	–	–
Weekend					
Yes	15 (34.9)	83 (21.8)	1.91 (0.98–3.74)	–	–
No	28 (65.1)	296 (77.9)	1.00	–	–
Unknown	0 (0.0)	1 (0.3)	–	–	–

bold indicates significant at 0.05 alpha level

*CI* confidence interval

^a^Peak traffic time defined as 6:00 am-8:59 am or 4 pm-5:59 pm on weekdays

^b^Adjusted for Driving Conditions, Intersection, Peak Time, Road Type and Traffic Control Device

^c^Adjusted for variables in ^b^as well as cyclist age and sex

## Discussion

Population level data from the two largest cities in Alberta, Canada, were used to identify bicyclist, driver, and environmental risk factors for severe injury in youth BMVCs. Using statistical/machine learning methods, this study found peak traffic time and divided roads with no physical barrier were associated with lower odds for severe injuries among children and adolescent bicyclists. It is possible that the road type itself is not reducing the risk of severe injury; however, these types of roads may be targets for traffic calming devices, lower speed limits, or lower traffic volumes. The protective effect of peak traffic times on severe injury from BMVCs may be partially due to slower vehicle speeds. As well, there may be more bicyclists travelling at this time leading to a “safety in numbers” effect (Jacobsen 2003), providing a potentially safer environment for bicyclists. Previous findings suggest that during school commuting hours, severe injuries in youth are less frequent than non-commuting hours (Mitchell et al. 2015).

Findings elsewhere suggest that children less than 15 years of age and males are at higher risk of severe bicycling injury (Martinez-Ruiz et al. 2013; Tin Tin et al. 2010). While relatively large (aOR = 1.82; 95% CI: 0.90–3.76), the odds for severe injury in males were not significantly higher than females in this study. Still, this study identified 81.3% of youth BMVCs involving male cyclists, potentially due to gender differences, where males simply have a greater exposure to active transportation (McMillan et al. 2006).

There is a paucity of research examining driver characteristics contribution to severe collision with youth cyclists; however, some risk factors for BMVCs in drivers include being over 60 years old or being under the influence of drugs or alcohol (Martinez-Ruiz et al. 2013). It was not possible to isolate drugs or alcohol in this study as there was only one collision where the driver was identified to be under the influence of alcohol and none under the influence of drugs. To this point, it is up to the officer’s assessment to determine whether or not the individual appeared to be under the influence at the time of the collision. Impaired by alcohol in this context is as follows: “In the judgment of the police officer, driving ability was impaired by alcohol consumption. Whether or not the subject was actually charged is not taken into consideration by the collision report form” (Alberta Transportation 2011).

Despite provincial legislation on helmets for youths, 49% of this sample were reported to not be wearing helmets. A previous Alberta-based study observed approximately 93% of under 13 year-olds and 63% of 14–17 year-olds wearing helmets when bicycling in an urban environment in 2006 (Karkhaneh et al. 2011), so it may be possible that overall helmet use has declined since legislation was passed in 2002. It has been previously suggested that those not wearing helmets would engage in less safe behavior, generally, thus leading to more severe injury collisions for the cyclist (Bambach et al. 2013). Therefore, it is also possible that the finding of fewer helmeted youth in these data represents youth who were more likely to not wear helmets and be involved in youth BMVCs.

Our study has limitations. First, while police collision reports can be a rich source of data, they do not contain factors such as measured speed of the vehicle/bicycle, cyclist experience, or cyclist information such as height or weight. Misclassification is possible, where those in our control group may have had their injuries progress in severity after the initial report; however, police reports are generally accurate at identifying severe injuries as defined in police collision reports (Sciortino et al. 2005). Underreporting, especially in cyclists and those under 19 years, is an issue with police report data (Watson et al. 2015) and it is plausible that this underreporting would be more prevalent in non-severe collision. This is a limitation of any collision dataset and future studies should seek to identify those who are not reporting BMVCs to evaluate how well those who do report represent the source population. A small sample size of 43 severe injuries was identified, limiting data analyses, the precision of estimate, and the ability to detect differences in estimates if such differences exist. Although these data represent five years of collisions from the two largest municipalities within the province of Alberta, the small sample size presents limitations in terms of statistical power and ability to develop complex regression models. This may also be why we fail to identify any confounding in the environmental effects when adjusting for child characteristics. Studies in larger jurisdictions or across jurisdictions may provide better precision by increasing sample size. Last, current police report structure may make it difficult for the officer to accurately identify primary event and pre-collision action for collisions involving bicyclists. Approximately 50% of the youth BMVCs were coded as “struck object” as the primary event; unfortunately, in the case of youth BMVCs this description provides little insight as to what specific actions contribute to severe injuries. This limitation of police reports and the need for improvement related to BMVC collisions has also been identified by the City of Calgary (2011).

## Conclusion

This study identifies protective factors for youth sustaining severe injury compared with those who did not after BMVCs, while considering both personal and environmental characteristics. Several environmental factors were identified that may contribute to decrease severity of injuries to youth bicyclists, indicating a greater need for interventions and research that focus on the environment in which these injuries occur. Moreover, this study suggests that future research is needed in this area where larger sample sizes are available.

## Additional file

Additional file 1: Exploration of characteristics of youth bicycle-motor vehicle collisions resulting in severe vs. non-severe injuries to cyclists using recursive partitioning. (PDF 57 kb)

## Abbreviations

aOR: Adjusted Odds Ratio
BMVC: Bicycle-Motor Vehicle Collision
CART: Classification and Regression Trees
ED: Emergency Department
OR: Odds Ratio

## References

[CR1] Alberta Transportation (2011). Alberta Traffic Collisions Statistics 2011.

[CR2] Bambach MR, Mitchell RJ, Grzebieta RH, Olivier J (2013). The effectiveness of helmets in bicycle collisions with motor vehicles: a case–control study. Accid Anal Prev.

[CR3] Barton BK, Morrongiello BA (2011). Examining the impact of traffic environment and executive functioning on children's pedestrian behaviors. Dev Psychol.

[CR4] Breiman L, Friedman JH, Olshen RA (1984). Classification and regression trees.

[CR5] Canadian Institute for Health Information (2017). Injury and Trauma Emergency Department and Hospitalization Statistics, 2015–2016.

[CR6] City of Calgary (2011). Cycling Strategy.

[CR7] City of Calgary (2013). Changing Travel Behaviour in the Calgary Region: Travel Report Behaviour Series Volume 1.

[CR8] City of Edmonton (2009). Cycle Edmonton: Bicycle Transportation Plan.

[CR9] City of Edmonton (2015). 2015 Edmonton and Region Household Travel Survey.

[CR10] de Hartog J, Boogaard H, Nijland H (2010). Do the health benefits of cycling outweigh the risks?. Environ Health Perspect.

[CR11] Embree TE, Romanow NTR, Djerboua MS, et al. Risk factors for bicycling injuries in children and adolescents: a systematic review. Pediatrics. 2016;138(5):e20160282. 10.1542/peds.2016-0282 27940760

[CR12] Government of Alberta (2012). Alberta Human Services: Alberta Awaits.

[CR13] Hagel BE, Romanow NT, Enns N (2015). Severe bicycling injury risk factors in children and adolescents: a case-control study. Accid Anal Prev.

[CR14] Jacobsen PL (2003). Safety in numbers: more walkers and bicyclists, safer walking and bicycling. Inj Prev.

[CR15] Jacobsen PL, Racioppi F, Rutter H (2009). Who owns the roads? How motorised traffic discourages walking and bicycling. Inj Prev..

[CR16] Karkhaneh M, Rowe BH, Saunders LD (2011). Bicycle helmet use four years after the introduction of helmet legislation in Alberta, Canada. Accid Anal Prev.

[CR17] Lehtonen E, Sahlberg H, Rovamo E (2017). Learning game for training child bicyclists' situation awareness. Accid Anal Prev.

[CR18] Martinez-Ruiz V, Lardelli-Claret P, Jimenez-Mejias E (2013). Risk factors for causing road crashes involving cyclists: an application of a quasi-induced exposure method. Accid Anal Prev.

[CR19] McMillan T, Day K, Boarnet M (2006). Johnny walks to school—does Jane? Sex differences in children's active travel to school. Children Youth Environ.

[CR20] Mitchell RJ, Bambach MR, Foster K (2015). Risk factors associated with the severity of injury outcome for paediatric road trauma. Injury.

[CR21] Prati G, Pietrantoni L, Fraboni F (2017). Using data mining techniques to predict the severity of bicycle crashes. Accid Anal Prev.

[CR22] R Core Team (2018). R: A language and environment for statistical computing.

[CR23] Romanow NT, Couperthwaite AB, McCormack GR (2012). Environmental determinants of bicycling injuries in Alberta, Canada. J Environ Public Health.

[CR24] Sciortino S, Vassar M, Radetsky M (2005). San Francisco pedestrian injury surveillance: mapping, under-reporting, and injury severity in police and hospital records. Accid Anal Prev..

[CR25] STATA Corp (2011). Statistical Software: Release 12.

[CR26] Statistics Canada. Focus on Geography Series, 2011 Census. Statistics Canada Catalogue no. 98-310-XWE2011004. Ottawa, Ontario. 2012. Available at: http://www12.statcan.gc.ca/census-recensement/2011/as-sa/fogs-spg/Facts-pr-eng.cfm?Lang=Eng&GK=PR&GC=48. Accessed January 10, 2019.

[CR27] Tin Tin S, Woodward A, Ameratunga S (2010). Injuries to pedal cyclists on New Zealand roads, 1988-2007. BMC Public Health.

[CR28] Watson A, Watson B, Vallmuur K (2015). Estimating under-reporting of road crash injuries to police using multiple linked data collections. Accid Anal Prev.

