# Supplementary material for: Child and adolescent bicycling injuries involving motor vehicle collisions
Source: Inj Epidemiol. 2019 Mar 4;6:7. doi: 10.1186/s40621-019-0185-z (PMC6582693; doi:10.1186/s40621-019-0185-z)
Supplement: Supplementary file 1 — Exploration of characteristics of youth bicycle-motor vehicle collisions resulting in severe vs. non-severe injuries to cyclists using recursive partitioning. (PDF 57 kb) [file 40621_2019_185_MOESM1_ESM.pdf]

```
n= 423

node), split, n, loss, yval, (yprob)
      * denotes terminal node

 1) root 423 43 0 (0.89834515 0.10165485)
   2) Sex=Female 79  4 0 (0.94936709 0.05063291) *
   3) Sex=Male 344 39 0 (0.88662791 0.11337209)
     6) Helmet=No 132 13 0 (0.90151515 0.09848485)
      12) Age.Group=13to17,7to12 115  9 0 (0.92173913 0.07826087) *
      13) Age.Group=0to6 17  4 0 (0.76470588 0.23529412)
        26) Driver.Action=FTY Uncontrolled,TCD Violation 2  0 0 (1.00000000 0
.00000000) *
        27) Driver.Action=Driving Properly 2  1 0 (0.50000000 0.50000000) *
     7) Helmet=Yes 212 26 0 (0.87735849 0.12264151) *
```

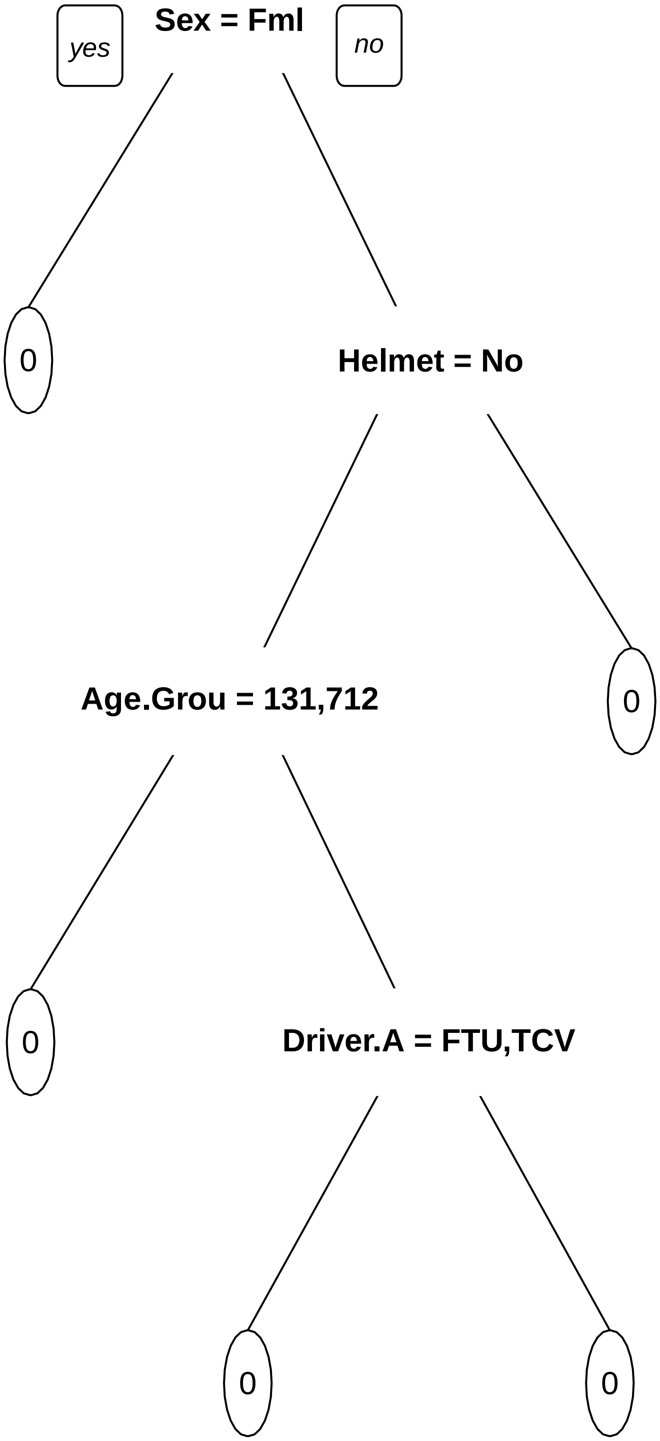

Sex = Fml
yes
no
0
Helmet = No
Age.Grou = 131,712
0
0
Driver.A = FTU,TCV
0
0

```
n= 423

node), split, n, loss, yval, (yprob)
      * denotes terminal node

 1) root 423 43 0 (0.89834515 0.10165485)
   2) Impact.Location=Left Side,Right Side 147  9 0 (0.93877551 0.06122449) *
   3) Impact.Location=Back,Front Centre 276 34 0 (0.87681159 0.12318841)
     6) Driver.Action=Driving Properly,FTY Uncontrolled,Lane Change or Turn,T
CD Violation 263 30 0 (0.88593156 0.11406844) *
     7) Driver.Action=Backed Unsafely 13  4 0 (0.69230769 0.30769231)
      14) Sex=Female 4  0 0 (1.00000000 0.00000000) *
      15) Sex=Male 9  4 0 (0.55555556 0.44444444)
        30) Age.Group=25to39,40to50 6  2 0 (0.66666667 0.33333333) *
        31) Age.Group=16to24,55to91 3  1 1 (0.33333333 0.66666667) *
```

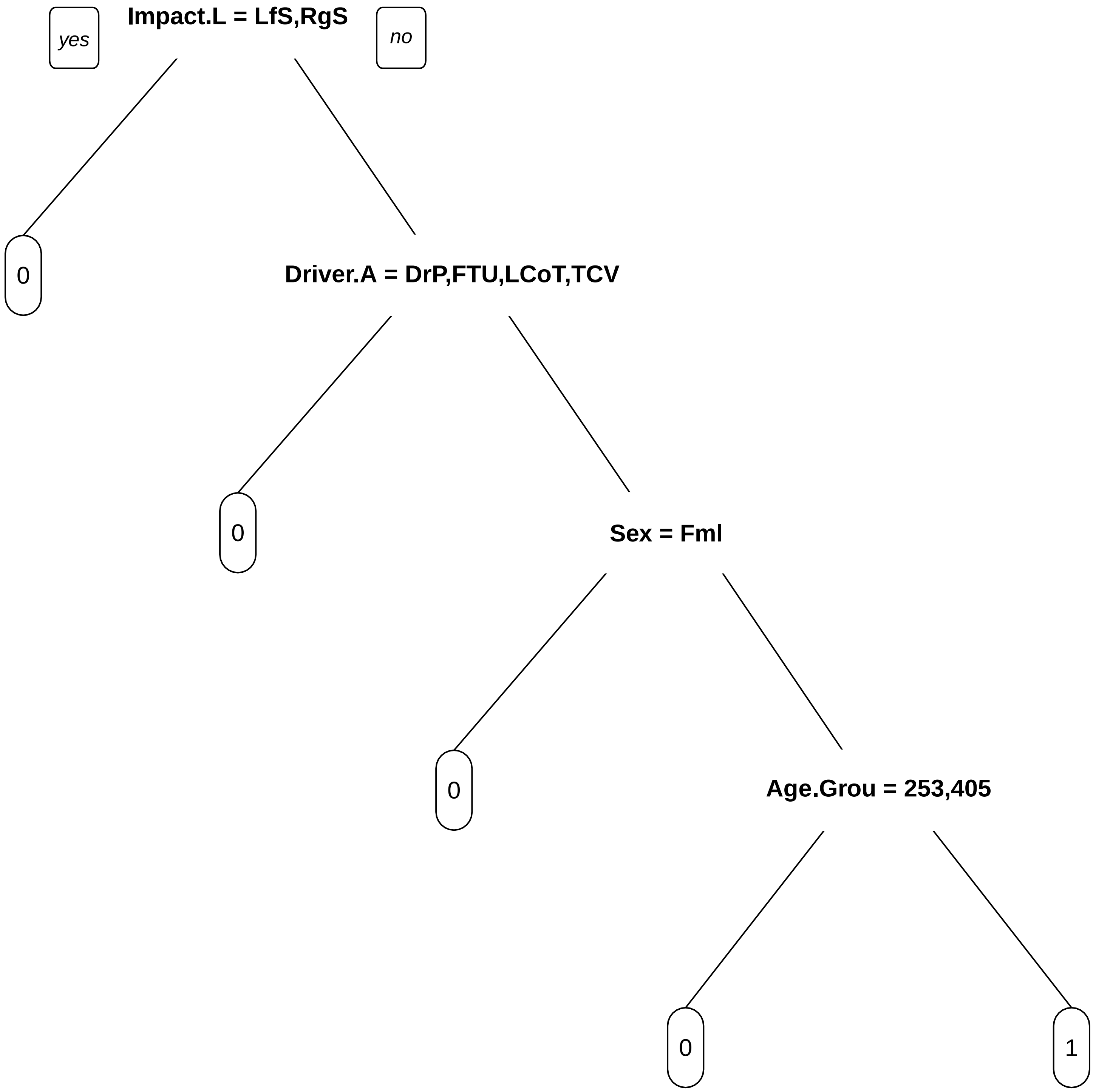
Impact.L = LfS,RgS
yes
no
0
Driver.A = DrP,FTU,LCoT,TCV
0
Sex = Fml
0
Age.Grou = 253,405
0
1

```
n= 423

node), split, n, loss, yval, (yprob)
      * denotes terminal node

 1) root 423 43 0 (0.89834515 0.10165485)
   2) tcds=Sign Present,Traffic Lights 181 12 0 (0.93370166 0.06629834) *
   3) tcds=Crosswalk,Nothing Present 242 31 0 (0.87190083 0.12809917)
     6) peaktimes=Yes 64  4 0 (0.93750000 0.06250000) *
     7) peaktimes=No 178 27 0 (0.84831461 0.15168539)
      14) roadtypes=Divided No Barrier,Other,Undivided Two-Way 133 16 0 (0.87
969925 0.12030075) *
      15) roadtypes=Divided With Barrier,Undivided One-Way 45 11 0 (0.7555555
6 0.24444444)
        30) tcds=Nothing Present 19  3 0 (0.84210526 0.15789474) *
        31) tcds=Crosswalk 26  8 0 (0.69230769 0.30769231)
          62) roadtypes=Divided With Barrier 23  6 0 (0.73913043 0.26086957)*
          63) roadtypes=Undivided One-Way 3  1 1 (0.33333333 0.66666667) *
```

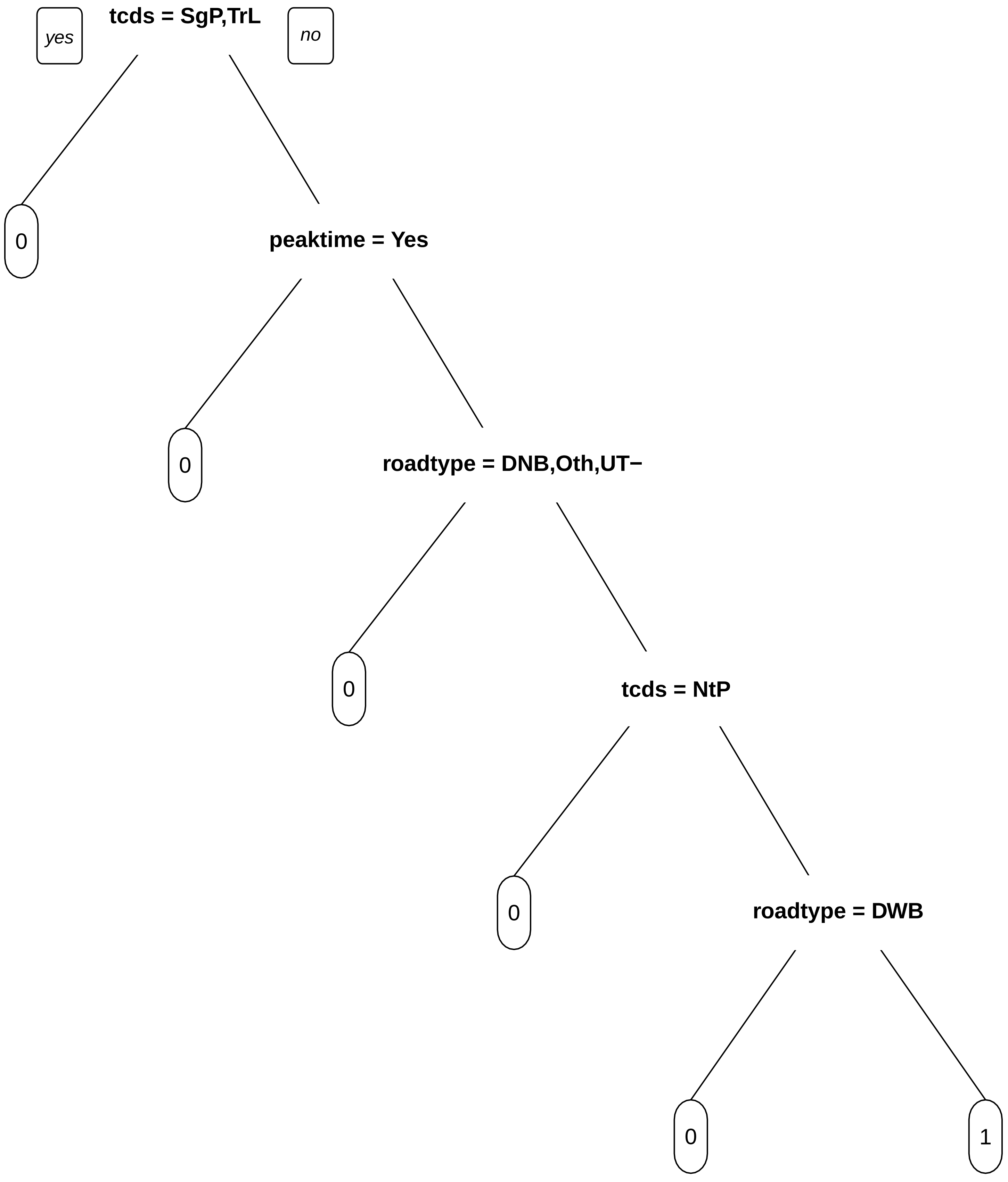
tcds = SgP,TrL
yes
no
0
peaktime = Yes
0
roadtype = DNB,Oth,UT−
0
tcds = NtP
0
roadtype = DWB
0
1
